# Supplementary material for: Osteoporotic hip fracture prediction from risk factors available in administrative claims data – A machine learning approach
Source: PLoS One. 2020 May 19;15(5):e0232969. doi: 10.1371/journal.pone.0232969 (PMC7237034; doi:10.1371/journal.pone.0232969)
Supplement: S1 File — (DOCX) [file pone.0232969.s001.docx]

# Electronic Supplementary Material

**Table S1** Algorithms included in Superlearner

| Algorithm | Description | R Package |
| --- | --- | --- |
| **Logistic regression with backward selection** | Parametric regression with a Logit Link to predict binary responses with a linear combination of predictor values. | Base |
| **Random Forest** with n=200 trees during the cv-stage and with n=1000 for the final model | An ensemble method that grows multiple trees on a bootstrapped sample of the training dataset. At each split, during the growths of the trees, a random subset of all possible predictors is used to avoid collinearity between predictions of the individual trees. | randomForest |
| **Support Vector Machine** with a Gaussian RBF kernel | Inputs vectors – the predictors – are mapped into a high dimensional feature space through either linear or non-linear kernel functions. This space is separated by a flat boundary called a hyperplane, which divides the space into homogeneous partitions. Classification depends on the side of the hyperplane a case belongs to. | e1071 |
| **RUSBoost with an undersampling ratio of 3:7 (minority class: majority class) and SVM with a linear kernel as weak learner** | RUSBoost is a hybrid approach that combines boosting and data sampling and designed to address imbalanced data sets. It combines random undersampling (RUS) and the AdaBoost algorithm (here: AdaBoost.M2). AdaBoost.M2 applies a weak learner (here: SVM with a linear kernel) to predict the most fitting category as well as a “degree of plausibility” for every assigned categorization. The importance of correctly classifying a case varies with each iteration and gradually increases for incorrectly classified cases which are particularly difficult to discriminate from other categories. Since AdaBoost.M2 focuses on these difficult cases it can outperform other methods in imbalanced datasets, where correctly classifying the minority class is often most challenging. | rusboost, adaboost |

**Extreme Gradient Boosting approach:**

We used the xgb.cv function in the xgboost library in R to obtain ideal parameters for our final model using 10-fold cross-validation on the training data. The xgboost library offers early stopping based on the area under the curve (AUC) for the hold out sample, which allows the model to stop the learning process if additional iterations (i.e. additional trees) have not lead to an improvement in the evaluation criterion (e.g. AUC or the classification error rate) for a certain number of rounds. We decided on n=50 rounds for the early stopping criteria. It is essential when using gradient boosting machines (GBM) to stop when the model does not improve on the hold-out sample, because otherwise the model is likely to be adapted too much to the training data and therefore does not generalize to the validation data set. Regarding additional parameters, we chose a learning rate of 0.01, which on average – over an applied grid search – constructed models that consisted of approximately n=450 trees. To find reasonable values for the vast set of parameters that control the learning process of GBMs, we used a grid search – i.e. we systematically applied 10-fold cross-validation to obtain test AUC values for different parameter constellations and chose one of the best model (several models performed similarly well). Table S2 lists the values we assessed during our grid search for the different GBM parameters. The final model, which was reported in the manuscript, was trained on the entire training sample and used the following settings: eta=0.01, max_depth=4, nrounds=450, gamma=0, alpha=0, lambda=0, colsample_bytree=0.5, subsample=0.8, min_child_weight=1.

**Table S2:** Set of values for GBM parameters assessed during the grid search

| Parameter | Values |
| --- | --- |
| **max_depth** | 2, 4, 6 |
| **min_child_weight** | 1, 3 |
| **subsample** | 0.8, 1 |
| **colsample_bytree** | 0.5, 1 |
| **gamma** | 0, 1, 10 |
| **alpha** | 0, 0.1, 1 |
| **lambda** | 0, 0.1, 1 |

**Model performance:**


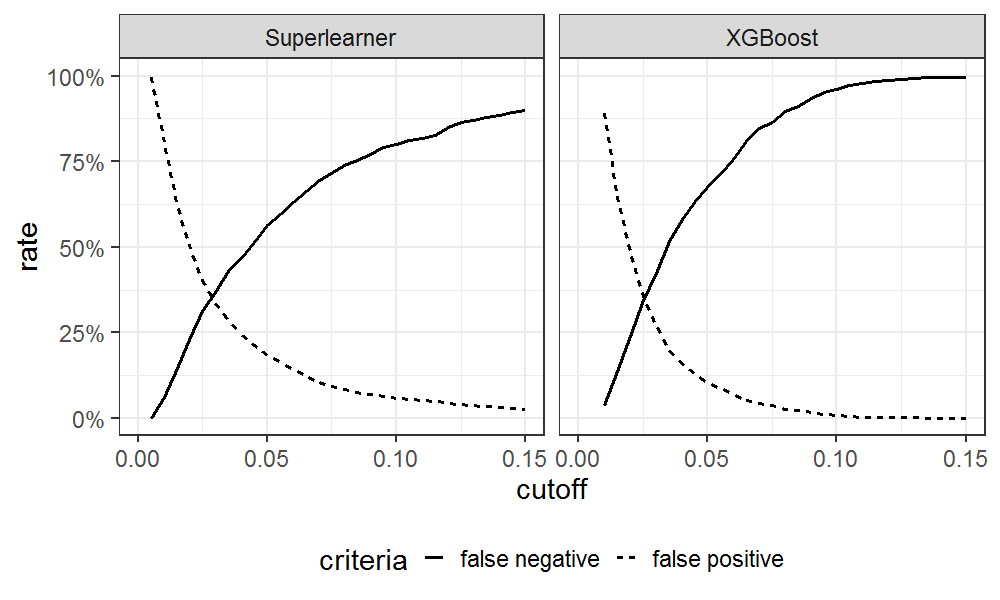


**Fig 1. False positive and false negative rate of XGBoost and the Superlearner.** The cutoff refers to the user-defined probability value necessary for the prediction model to classify patients as “at risk” for the fracture event. The false positive rate refers to the proportion of all non-fracture patients that are incorrectly classified as “at risk” for the event. The false negative rate refers to the proportion of all patients who will have a hip fracture within the observation period, but are incorrectly classified as “not at risk” for the event.

Both the false positive and the false negative rate depend on the so called cutoff – i.e. the required probability value for the prediction of a fracture event. The false positive rate refers to the proportion of all non-fracture patients that are incorrectly classified as “at risk” for the event. If the cutoff is relatively small, a lot of insurees are classified as “at risk”, therefore the rate of false positives is relatively high. Complementarily, the false negative rate refers to the proportion of all patients who will have a hip fracture within the observation period, but are incorrectly classified as “not at risk” for the event. If the cutoff is relatively small, more patients are classified as “at risk”, therefore the false negative rate  i.e. the risk of falsely classifying someone as “not at risk” is small.


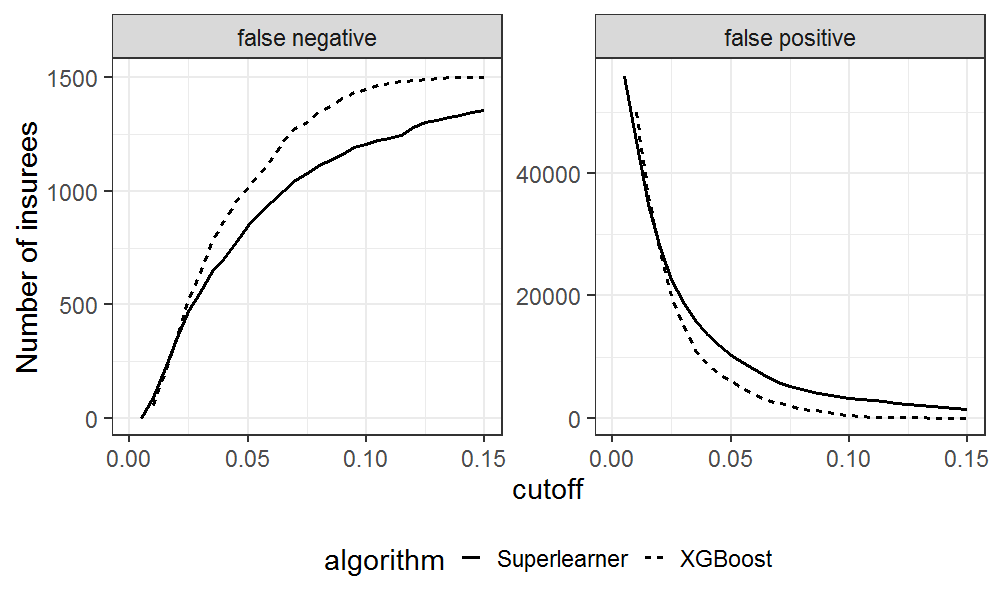


**Fig 2. Number of false positive and false negative insurees under XGBoost and the Superlearner.** The cutoff refers to the user-defined probability value necessary for the prediction model to classify patients as “at risk” for the fracture event. The number of false positives refers to the number of patients that are incorrectly classified as “at risk” for the event in the validation dataset (N=57,618). The number of false negatives refers to the number of patients who will have a hip fracture within the observation period, but are incorrectly classified as “not at risk” for the event in the validation dataset.


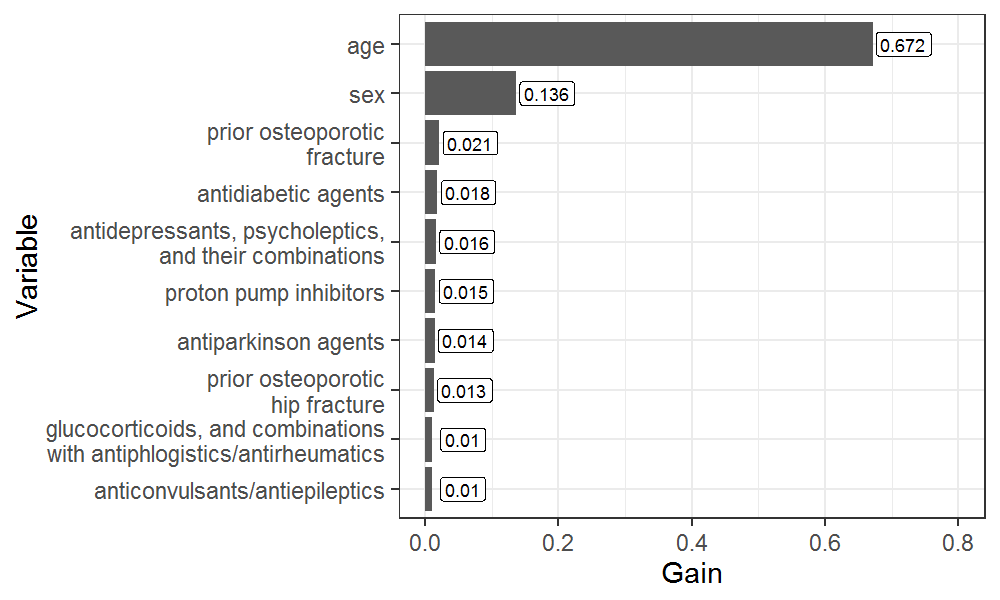


**Fig 3. Variable Importance Plot for the extreme gradient boosting model.**

The variable importance plot depicts how much each feature contributes to the gain in the evaluation criterion by splitting the original sample into smaller groups according to a decision rule based on that feature (e.g. age > 65 years) during the tree growing process. Higher values indicate that the feature is relatively (i.e. when compared to the other features) important for the prediction of the outcome. However, the absolute values convey no meaning by themselves. Consequently, the plot can mainly be used to distinguish predictive from less predictive features, but it can’t be used to assess the utility of the model or the general predictive performance.
